# Sensitivity analysis of human brain structural network construction

**DOI:** 10.1162/NETN_a_00025

**Published:** 2017-12-01

**Authors:** Kuang Wei, Matthew Cieslak, Clint Greene, Scott T. Grafton, Jean M. Carlson

**Affiliations:** Department of Physics, University of Chicago, Chicago, IL, USA; Department of Physics, University of California, Santa Barbara, CA, USA; Department of Psychological and Brain Sciences, University of California, Santa Barbara, CA, USA; Department of Electrical and Computer Engineering, University of California, Santa Barbara, CA, USA

**Keywords:** Tractography, Brain networks, Human Connectome Project, Connectomics, White matter connectivity

## Abstract

Network neuroscience leverages diffusion-weighted magnetic resonance imaging and tractography to quantify structural connectivity of the human brain. However, scientists and practitioners lack a clear understanding of the effects of varying tractography parameters on the constructed structural networks. With diffusion images from the Human Connectome Project (HCP), we characterize how structural networks are impacted by the spatial resolution of brain atlases, total number of tractography streamlines, and grey matter dilation with various graph metrics. We demonstrate how injudicious combinations of highly refined brain parcellations and low numbers of streamlines may inadvertently lead to disconnected network models with isolated nodes. Furthermore, we provide solutions to significantly reduce the likelihood of generating disconnected networks. In addition, for different tractography parameters, we investigate the distributions of values taken by various graph metrics across the population of HCP subjects. Analyzing the ranks of individual subjects within the graph metric distributions, we find that the ranks of individuals are affected differently by atlas scale changes. Our work serves as a guideline for researchers to optimize the selection of tractography parameters and illustrates how biological characteristics of the brain derived in network neuroscience studies can be affected by the choice of atlas parcellation schemes.

## Author Summary

Diffusion tractography has been proven to be a promising noninvasive technique to study the network properties of the human brain. However, how various tractography and network construction parameters affect network properties has not been studied using a large cohort of high-quality data. We utilize data provided by the Human Connectome Project to characterize the changes to network properties induced by varying the brain parcellation atlas scales, the number of reconstructed tractography tracks, and the degree of grey matter dilation with graph metrics. We illustrate the importance of increasing the reconstructed track sampling rate when higher atlas scales are used. In addition to changing the raw values of graph metrics, we find that the ranks of individuals relative to the population metric distributions are altered. We further discuss how the dependency of graph metric ranks can affect the brain characteristics derived in group comparison studies using network neuroscience techniques.

## Introduction

The human connectome, the structural connectivity map of the human brain, was proposed in 2005 to further understanding of how cognitive processes emerge from an underlying structural substrate (Sporns, Tononi, & Kötter, 2005). The structural connectivity map models the grey matter regions and the white matter pathways connecting them to form a complex network. This approach enables quantitative studies to characterize neurological processes using theoretical graph analysis. Previous studies have found whole-brain networks to exhibit qualities such as small-worldness, rich club organization, network hierarchy, and other features (Achard, Salvador, Whitcher, Suckling, & Bullmore, 2006; Bassett et al., 2010; Bullmore & Sporns, 2009; Collin, Sporns, Mandl, & van den Heuvel, 2014). Certain topological features of the brain structural network have been linked to brain disorders, suggesting the possibility of utilizing network models and graph analysis in clinical diagnosis (Crossley et al., 2014).

Given the promising power of whole-brain network modeling, the Human Connectome Project (HCP) was designed to enable comprehensive studies of the human brain structural circuitry and its relationship to cognitive functions by freely providing high-quality structural neuroimaging data for a large population of individuals (Essena, 2012; Toga, Clark, Thompson, Shattuck, & Van Horn, 2012; Van Essen et al., 2013). However, the extent to which these graph analysis results are affected by tractography parameters, algorithms, and network construction methods remains to be quantified in a large cohort of high-quality data. Before graph analysis can be widely adopted in clinical diagnosis, an understanding of the sensitivity of human brain networks to tractography parameters is necessary. The large sample size of the HCP enables us to identify the statistically significant effects of tractography parameters on constructed networks and develop robust methods to construct brain structural connectivity networks.

The starting point for modeling the brain as a complex network involves parcellating cortex (and potentially subcortical areas) into a set of nonoverlapping regions, which serve as nodes, and choosing one of the available methods for weighting interregional connectivity using diffusion-weighted imaging (DWI) tractography to serve as edge weights (Iturria-Medina, Sotero, Canales-Rodriguez, Alemán-Gómez, & Melie-García, 2008). However, the process in which brain structural images are converted into graphs has not yet been standardized. One of the critical steps in this process is to select a brain atlas. Some atlases are specific to an individual’s anatomy. For example, FreeSurfer-based atlases such as the Desikan-Killiany atlas define regions based on each brain’s gyri (Desikan et al., 2006). Other atlases are defined on standardized brain templates (such as the Automated Anatomical Labeling Atlas and the LONI Probabilistic Brain Atlas) and are typically registered to an individual’s brain with an affine or nonlinear transform (Shattuck et al., 2008; Smith et al., 2004; Tzourio-Mazoyer et al., 2002; Woods, Grafton, Watson, Sicotte, & Mazziotta, 1998). Although some atlases incorporate multiple atlas spatial resolutions/scales (to avoid terminology confusion, atlas spatial resolutions will be worded as atlas scales in this paper) (Daducci et al., 2012), many have fixed atlas scales and the number of regions of interest can range from below 100 to greater than 10^3^ Gong et al., 2009; Hagmann et al., 2007; Iturria-Medina, Canales-Rodriguez, et al., 2007; Iturria-Medina, Sotero, et al., 2008; Zalesky et al., 2010).

Similarly, numerous methods exist for generating edge weights. A common measure, streamline count, is based on indirect measurements of neuronal connections constructed according to the preferred directions of water diffusion in axonal tissues (Tuch, 2004). However, streamlines are known to be inaccurate in representing the axon density between two regions (Jones, Knösche, & Turner, 2013). They also rely on fiber-tracking algorithms that have been shown to exhibit systematic inaccuracy across large areas of the brain (Fallani, Richiardi, Chavez & Achard, 2014; Knösche, Anwander, Liptrot, & Dyrby, 2015; Reveley et al., 2015; van denHeuvel & Pol, 2010). Nevertheless, streamline count remains widely used as an edge weight.

These tractography parameter choices have been shown to impact the topological properties of the networks constructed from DWI (Bassett, Brown, Deshpande, Carlson, & Grafton, 2011; Zalesky et al., 2010). As scientific conclusions derived from graph analysis may be significantly impacted by tractography algorithms and parameters, these findings must be assessed in the context of the chosen network construction methods. In this study, we use graph metrics and summary statistics to quantify the extent to which the choices of brain atlas scales, grey matter dilation, and streamline count impact brain network models and their topological properties. In addition, we also analyze how the relative ranks of individuals in the population graph metric distributions change with varying tractography parameter settings. The rank comparison reveals that changes in tractography parameter values introduce inhomogeneous changes to the network properties of each individual, instead of universally shifting the graph metric values of individuals. The key findings are as follows.

(a)  Structural connectivity networks constructed in high atlas scales may contain disconnected nodes. Increasing streamline sampling rate and dilating grey matter significantly reduce the likelihood of generating disconnected nodes.
(b)  Increase in streamline count and grey matter dilation both raise network density, though they impact network topology differently.
(c)  The spatial resolution of the atlas scales imposed has a significant effect on the topology of the constructed networks. Streamline count and grey matter dilation have smaller effects on graph metric values.
(d)  Atlas scales also change how grey matter dilation alters network topology.
(e)  Brain atlas scales affect the absolute value of many network metrics significantly, and individual subjects respond differently. Therefore, a change of atlas scale can significantly alter the rank of a network within the population metric distribution.
(f)  Streamline count and grey matter dilation on average do not change the rank of a subject significantly in the population distribution.

These findings support the need to account for variations in tractography parameters in order to compare brain network characteristics across individuals and studies.

## METHODS

### Data and Preprocessing

The dataset was collected as part of the Washington University–Minnesota Consortium Human Connectome Project (Van Essen et al., 2013). Participants were recruited from Washington University (St. Louis, Missouri) and the surrounding area. All participants gave informed consent. The data used were from the S500 release, consisting of structural and diffusion data from 489 participants. The structural and diffusion data were collected on 3T Connectome Skyra system (Siemens, Erlangen, Germany) at various spatial and angular resolutions. The scanner was equipped with SC72 gradients operating at 100 mT/m maximum gradient amplitude with a maximum slew rate of 91 T/(m × s) for improved diffusion encoding. The diffusion volumes were collected with a spatial resolution 1.25×1.25×1.25 mm^3^, using three shells at b = 1,000, 2,000, and 3,000 s/mm^2^ with 90 diffusion directions per shell and 10 additional b0s per shell. Among the 489 participants, 49 of them have either 30 or 60 diffusion directions per shell. We select 440 participants with a full set of 90 diffusion directions as our subjects for this study. The list of 49 subjects omitted in this study is included in the Supporting Information (Wei, Cieslak, Greene, Grafton, & Carslon, 2017). The diffusion data were corrected for geometric and eddy current distortions, using information from acquisitions in opposite phase-encoding directions, as well as head motion (Glasser et al., 2013). The gradient table for these images follow the protocol proposed in Caruyer, Lenglet, Sapiro, and Deriche, (2013). The high-resolution structural T1w and T2w volumes were acquired on the same scanner using a 32-channel head coil at 0.7 mm isotropic resolution (Glasser et al., 2013).

### Data Reconstruction

HARDI diffusion datasets were reconstructed in DSI Studio using GQI with a mean diffusion distance of 1.25 mm with up to five fiber orientations per voxel (Yeh, Wedeen, & Tseng, 2010). Fiber tracking was performed in DSI Studio with an angular cutoff of 60°, step size of 0.675 mm, minimum length of 10 mm, smoothing of 0.0, and maximum length of 420 mm. Along with the angular cutoff and minimum and maximum fiber length, additional stopping criteria included a tracking mask with the threshold for trackable voxels set to 0.6 × Otsu threshold of the quantitative anisotropy (QA) image. QA is an anisotropy index similar to fractional anisotropy/generalized fractional anistropy (FA/GFA), but is calculated for each ODF peak in each voxel. QA enables direction-specific thresholding during tractography, and therefore is less susceptible to partial volume effects, less noisy, and advantageous to improving tractography performance (Yeh, Verstynen, Wang, Fernández-Miranda, & Tseng, 2013). These reconstruction parameters are standard and well-performing parameters for the DSI Studio deterministic tracking pipeline (Maier-Hein et al., 2016). An improved and top-performing (ISMRM 2015 Tractometer Challenge) deterministic fiber-tracking algorithm was used until either 10^4^, 10^5^, or 10^6^ streamlines were reconstructed for each subject (Yeh et al., 2013). Note that the number of streamlines is determined by the user during streamline reconstruction and is not dependent on the tractography algorithm itself. Whole-brain subvoxel seeding with tracking started in the direction of the primary fiber orientation was conducted until the specified number of streamlines was generated. A pseudorandom number generator was used to place the seeds, and the seed distribution was deterministically random. This ensured that the same seeding sequence was used and that the tracking result was reproducible using the same tracking parameters. In addition, we also separately performed tractography with cortical and subcortical region labels projected 2.5 mm into white matter to account for streamlines that were not tracked through to grey matter. The same atlas dilation technique used by Cieslak and Grafton 2014 was employed. Datasets reconstructed with this grey matter dilation setting are labeled as dilation two, or D2, and the rest of the datasets without grey matter dilation are labeled as D0.

T1w anatomical scans were segmented using FreeSurfer and parcellated according to the Lausanne 2008 atlas included in the connectome mapping toolkit (Daducci et al., 2012; Fischl & Dale, 2000; Hagmann, et al., 2008). The atlas uses the standard FreeSurfer subcortical segmentation, which includes 8 regions (thalamus proper, caudate, putamen, pallidum, accumbens area, hippocampus, amygdala, and brainstem). Four parcellation schemes, Scale 33 (containing 83 regions), Scale 60 (129 regions), Scale 125 (234 regions), and Scale 250 (463 regions), were registered to the b0 volume from the HARDI data each subject. The spatial resolution of the subcortical nodes does not change with the cortical atlas scale. The approximate volume of each region of interest of the Lausanne atlas is kept consistent in order to prevent improper bias towards certain regions when constructing streamlines (Daducci et al., 2012). The atlases were defined on each subject’s cortical surface so no nonlinear registration was necessary. Among the 440 subjects, 22 subjects’ BBRegister failed to accurately coregister the FreeSurfer parcellation to the b0 DWI scan. The 22 subjects are removed from this study.

### Network Construction

In this study, we distinguish between weighted and unweighted networks. A weighted network is represented by a weighted connectivity matrix, **W**. The entry *W*_*ij*_ represents the total number of streamlines connecting nodes that correspond to cortical regions *i* and *j*. For example, a weighted connectivity matrix produced in the Scale 33 atlas is an 83×83 matrix. An unweighted network disregards the number of streamlines and only establishes edges based on whether at least one streamline exists between a pair of nodes. The connectivity matrix of an unweighted network, **U**, can be obtained by binarizing **W**. A nonzero entry, *U*_*ij*_, represents the existence of at least one streamline connecting cortical regions represented by nodes *i* and *j*.

### Network Characterization

In this study, there are several graph metrics that are calculated for both unweighted and weighted networks. Graph metrics computed for unweighted networks are referred to as unweighted metrics, and graph metrics computed for weighted networks are referred to as weighted metrics.

We compute various graph metrics to quantify the dependency of network characteristics on tractography parameters and network construction methods. The specific tractography and network construction parameters that we consider are the total number of streamlines in a network (streamline count, SC 10^4^, 10^5^, or 10^6^), grey matter dilation (D0 or D2), brain atlas scale (Scale 33, 60, 125, and 250), and streamline edge weighting. For each subject, graph metrics are calculated for all the networks constructed using varying tractography and network construction parameters. The population distributions of these metrics are reported and we focus on not only the values of the metrics of a network, but also the ranks of a network in the population distributions.

#### Density.

Network density is computed for unweighted networks, and is defined as

$$\rho =\frac{2M}{N(N-1)},$$ (1)

where $M=\frac{1}{2}\sum_{i,j}U_{ij}$ is the total number of edges and *N* is the total number of nodes in a connectivity network. An unweighted definition of network density, where the strengths of connections are disregarded, is appropriate since the goal of calculating the network density is to provide a quantitative measurement of the sparsity of a given connectivity network.

#### Minimum path length.

The minimum path length, *P*_*ij*_, between nodes *i* and *j* is defined as the minimum number of edges required to travel from node *i* to node *j* (Dijkstra, 1959). We compute the path length matrix **P** with entries *P*_*ij*_ to show the minimum path length between every pair of nodes in a connectivity network.

If the minimum path length between a pair of nodes is small, then the connection can be interpreted as efficient. Although not a focus of this study, the average minimum path length of all pairs of nodes in a network has been used to quantify the overall efficiency of a network (Toga et al., 2012).

#### Assortativity.

Assortativity can be calculated for both unweighted and weighted networks. In a unweighted network, a node degree, $k_{i}=\sum_{j}U_{ij}$, is defined as the number of edges emanating from node *i*. Assortativity quantifies the correlation between the degrees of individual nodes and the degrees of the nodes to which they are connected. Unweighted assortativity, *α*, is computed by summing over all edges in a network and is defined as

$$\alpha =\frac{M^{-1}\sum_{m}j_{m}k_{m}-{\left[M^{-1}\sum_{m}\frac{1}{2}(j_{m}+k_{m})\right]}^{2}}{M^{-1}\sum_{m}\frac{1}{2}(j_{m}^{2}+k_{m}^{2})-{\left[M^{-1}\sum_{m}\frac{1}{2}(j_{m}+k_{m})\right]}^{2}},$$ (2)

where *M* is the total number of edges in a network and *j*_*m*_, *k*_*m*_ are degrees of the nodes on either end of the *m*th edge (Newman, 2002). If a network has a positive assortativity, on average nodes are connected to other nodes with similar degrees. If the assortativity is negative, nodes with dissimilar degrees will more likely be connected. The absolute value of assortativity reflects the strength of the node degree correlations. Weighted assortativity, *α*_*w*_, is a generalized form of Equation 2 for weighted networks, where *α*_*w*_ is calculated by replacing the degree counts *j*_*m*_ and *k*_*m*_ by node strengths, *u*_*m*_ and *w*_*m*_, with *w*_*m*_ = $\sum_{n}W_{mn}$.

The unweighted assortativity measures the likelihood of a highly connected node to be connected to other highly connected nodes, regardless of the strengths of the connections. The weighted assortativity describes the likelihood of the neighboring nodes of a strongly connected node to also be strongly connected. With this definition, an assortative unweighted network may simultaneously be classified as a disassortative weighted network, or vice versa. For unweighted metrics, all present connections contribute equally, while for weighted metrics, edges with high streamline counts heavily influence the node degree correlation.

#### Modularity.

We compute the unweighted modularity of a network using the Louvain community detection algorithm to maximize the modularity quality function:

$$Q=\frac{1}{2M}\left[\right.\underset{ij}{\sum }\left(\right.U_{ij}-\gamma \frac{k_{i}k_{j}}{2M})\delta (g_{i},g_{j})],$$ (3)

where *U*_*ij*_ is the unweighted connectivity matrix, *γ* is the resolution parameter, *k*_*i*_ is the degree of node *i*, *M* is the total number of edges with $M=\frac{1}{2}\sum_{ij}U_{ij}$, and *δ* is the Kronecker delta with *δ*(*g*_*i*_, *g*_*j*_) = 1 if *g*_*i*_ = *g*_*j*_ and 0 otherwise (Fortunato, 2010; Newman, 2004b, 2006; Newman & Girvan, 2004; Porter, Onnela, & Mucha, 2009). Inside the Kronecker delta, *g*_*i*_ denotes that node *i* is in community *g*_*i*_. The term $\frac{k_{i}k_{j}}{2M}$ is the expected likelihood of the edge connecting node *i* to node *j* under the Newman-Girvan null model (Newman & Girvan, 2004). In our analysis, we set the resolution parameter, *γ*, to 1 by default.

Modularity can also be generalized for weighted networks (Newman, 2004a). Weighted modularity is defined as

$$Q_{w}=\frac{1}{2M}\left[\right.\underset{ij}{\sum }\left(\right.W_{ij}-\gamma \frac{w_{i}w_{j}}{2M})\delta (g_{i},g_{j})].$$ (4)

Here *W*_*ij*_ is the weight of an edge connecting node *i* and node *j*, and *M* has the same definition as in Equation 3. In the generalized weighted modularity equation, node degrees are replaced by node strengths *w*_*i*_ = $\sum_{j}W_{ij}$ and the term $\frac{w_{i}w_{j}}{2M}$ is again the expected weight of the edge connecting node *i* to node *j* in a randomized network.

We use a multi-iterative approach to reach the optimal community structure where sub divisions of the network are nonoverlapping groups of nodes that maximize the number of within-group edges and minimize the number of between-group edges (Blondel, Guillaume, Lambiotte, & Lefebvre, 2008; Bullmore & Sporns, 2009; Reichardt & Bornholdt, 2006; Ronhovde & Nussinov, 2009; Sun, Danila, Josi, & Bassler, 2009). Modularity by definition is restricted to values between –1 and 1. Networks with dense connections between nodes within their own communities but sparse connections between nodes outside of the modules that they belong to have high modularity. Biological networks have been shown to have high modularity (Newman, 2006).

#### Clustering coefficient.

Like assortativity and modularity, clustering coefficient can be calculated for both unweighted and weighted networks. For an unweighted network, the local clustering coefficient of each node *i*, *C*_*i*_ is defined as

$$C_{i}=\frac{\Delta_{exist}}{\Delta_{possible}},$$ (5)

where *Δ*_*exist*_ denotes the number of triangle subgraphs that include node *i*, and *Δ*_*possible*_ is the number of possible triplets that contains node *i* (Watts & Strogatz, 1998). The clustering coefficient of a network is calculated by averaging the local clustering coefficients of all the nodes in a network.

The unweighted local clustering coefficient can be generalized and used for weighted networks to calculate the weighted local clustering coefficient ${\tilde{C}}_{i}$, with

$${\tilde{C}}_{i}=\frac{1}{w_{i}(k_{i}-1)}\underset{j,h}{\sum }\frac{\left(W_{ij}+W_{ih}\right)}{2}U_{ij}U_{ih}U_{jh},$$ (6)

where the factor *w*_*i*_(*k*_*i*_ − 1) normalizes ${\tilde{C}}_{i}$ to ensure that $0\leq {\tilde{C}}_{i}\leq 1$. With this definition, ${\tilde{C}}_{i}$ fulfills the requirement that ${\tilde{C}}_{i}\to C_{i}$ when edge weights are binarized (Barrat, Barthelemy, Pastor-Satorras, & Vespignani, 2004).

The unweighted clustering coefficient characterizes the tendency of the nearest neighbors of a node to be interconnected. The weighted clustering coefficient accounts for edge weights and quantifies the likelihood of nodes with high strengths (based on the total number of streamlines emnating from the node) to cluster with other strong nodes. If the weighted clustering coefficient of a network is higher than the corresponding unweighted value, triplets are more likely formed by edges with high weights.

### Graph Metrics Calculation

The graph metrics reported in this study are all calculated with the Brain Connectivity Toolbox (BCT) implemented in MATLAB R2012a (Rubinov & Sporns, 2010). The BCT is a comprehensive toolbox for complex network analysis of sturctural and functional brain connectivity networks. This toolbox is especially suitable for this study since it provides both unweighted and weighted variants for all of the included graph metric measures, a feature not commonly available in other software packages. All of the network metrics included in the toolbox and the definitions of the various data input types that it processes are discussed in the 2010 article by Rubinov and Sporns (Rubinov & Sporns, 2010). The BCT is freely available at http://www.brain-connectivity-toolbox.net/.

## RESULTS

As described in Methods, for each subject we construct a total of 48 weighted and unweighted structural brain networks consisting of different brain atlas scales, total numbers of streamlines, and grey matter dilation settings. We calculate weighted and unweighted graph metrics for each of the constructed network of every subject and present the corresponding population metric distributions. Our purpose is to quantify the intrinsic variability of metrics across the population and the sensitivity of each metric to tractography and network construction parameters. Distinct from prior studies, we analyze the variability by illustrating the changes in both the raw values of the metrics and the relative ranks of individuals through examining the changes of individual ranks in the population distributions.

Note that although we consider three variations of SC (SC 10^4^, 10^5^, and 10^6^) to cover a larger portion of the SC parameter space, the overall effects of SC on graph metrics population distributions that we observe with SC 10^5^ and 10^6^ are generally consistent with what we find for SC 10^4^. To avoid cluttering figures, nclude the analysis from SC 10^5^ and 10^6^ in the main text and the results from SC 10^4^ in the Supporting Information (Wei et al., 2017). In addition, networks generated with SC 10^4^ are found to have problems associated with severe streamline undersampling and are unfit to be included in the main text.

### Density

**Figure 1.**
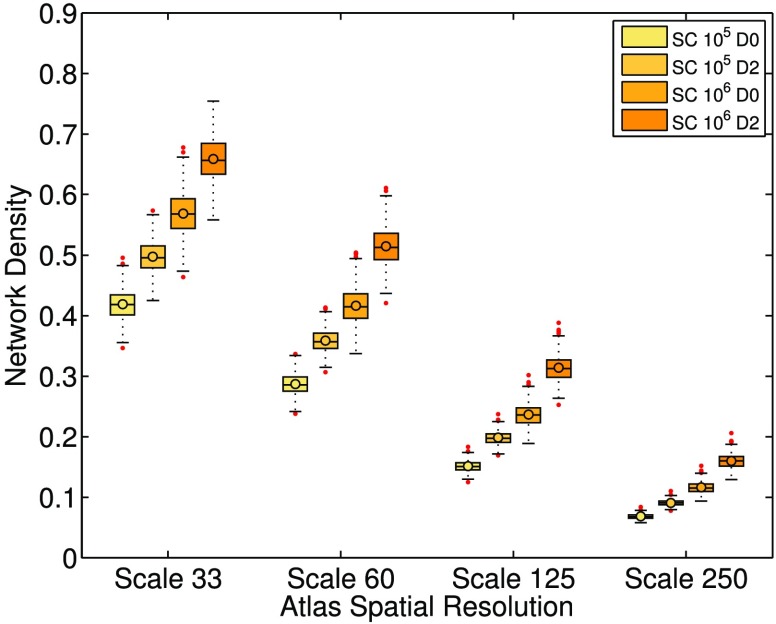
Network density population distributions with varying atlas scales, dilation, and streamline count. The labels SC 10^5^ and 10^6^ denote datasets constructed under the tractography setting of a total streamline count of 10^5^ or 10^6^, respectively. The labels D0 and D2 denote datasets constructed without or with grey matter dilation, respectively. The upper and lower bounds of the boxes represent the 25th and 75th percentile of the distribution, and the line in the boxes represents the median, while the circle represents the average. The two horizontal lines below and above the box are marked by 1.5 times of the interquartile range outside of the first and third quartile, respectively. Data points outside of this range are deemed outliers and are marked as red points. The rest of the box plots in this study follow the same notations. Overall, networks with SC 10^6^ and D2 have higher network density. This result is expected since both high streamline count and grey matter dilation introduce a higher probability of grey matter region pairs being connected by streamlines. In contrast, the density decreases with increasing atlas scales.

Network density is defined to be the number of connections in the unweighted network divided by the number of possible connections. Figure 1 illustrates the effects of atlas scale, grey matter dilation, and streamline count on the population distributions of network density. The networks of all subjects exhibit decreasing density as the spatial resolution of the brain atlas increases. Across the population, network density increases when grey matter regions are dilated. The increase is expected since grey matter dilation allows streamlines to reach grey matter more easily and form more connections between region pairs. We also see an increase in density across the population when the total number of streamlines is increased from 10^5^ to 10^6^, indicating that the addition of streamlines is not merely providing redundant measurements.

**Figure 2.**
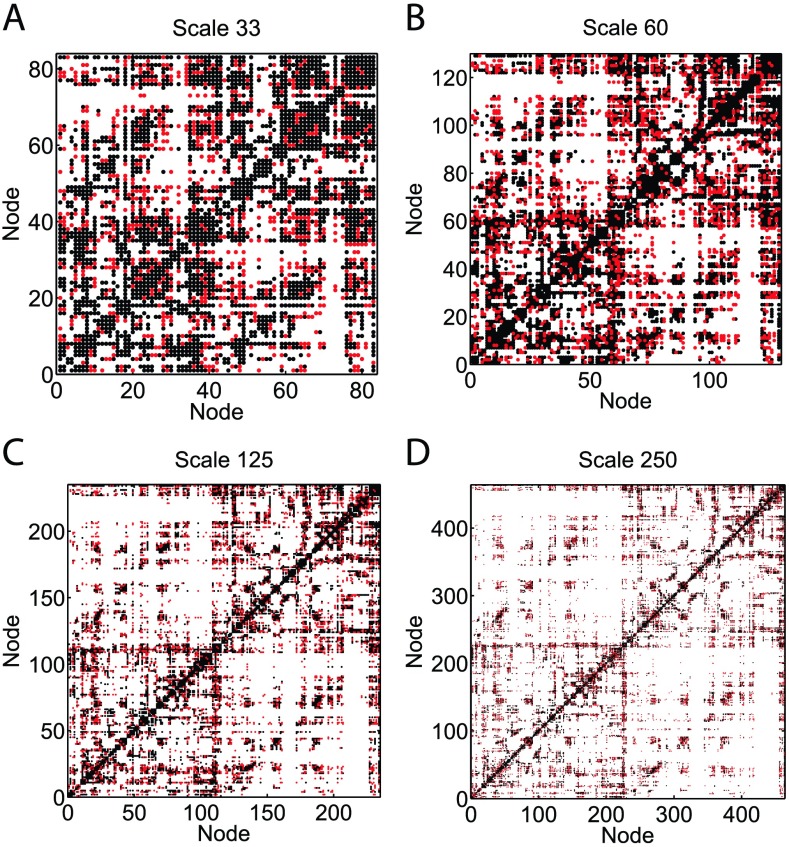
Comparison of the unweighted connectivity networks of a representative subject constructed with SC 10^5^ and 10^6^. The black points represent edges constructed using SC 10^5^, and the red points are the new edges constructed using SC 10^6^. The increase in the percentage of new edges is more significant as the atlas scales become higher (each node represents a more refined and smaller region). Table 1 summarizes the percentage increase in the number of edges formed when the SC is increased from 10^5^ to 10^6^ for this subject.

Figure 2 compares the unweighted connectivity matrices **U** for a representative subject constructed with SC 10^5^ (black) and SC 10^6^ (black + red) at every atlas scale. Table 1 summarizes the percentage increase in the number of new connections for this particular subject. The percentage increase is significant at every scale and is as high as 65.5% at Scale 250, suggesting that sampling 10^5^ streamlines is largely incomplete.

**Table 1.** Total number of edges in the unweighted connectivity networks of a representative subject. The corresponding connectivity networks are illustrated in Figure 2. The percentage of new edges that are constructed when the total streamline count increases from 10^5^ to 10^6^ is significant at every scale. The percentage increase is the highest, 65.5%, when the network is constructed at Scale 250. This suggests that a streamline sampling rate of 10^5^ significantly undersamples the network.

**Streamline Count**	**Scale 33**	**Scale 60**	**Scale 125**	**Scale 250**
10^5^	1,380	2,243	3,892	6,851
10^6^	1,823	3,163	5,887	11,341

**% increase in number of edges**	32.1%	41.0%	51.3%	65.5%

### Minimum Path Length

**Figure 3.**
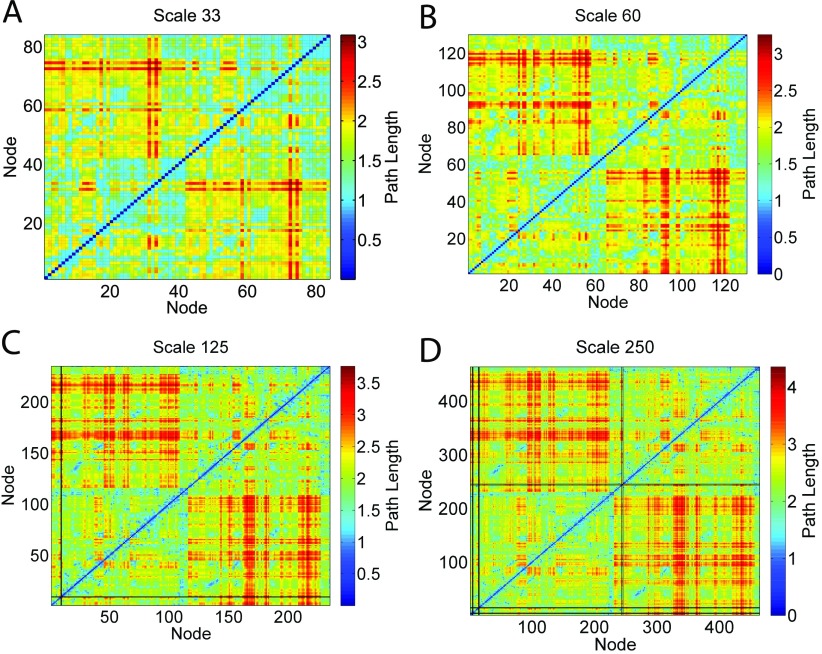
Population average path length matrices, **A**, at four atlas scales. The black lines are infinite path length lines, which represent nodes that are disconnected from the giant component of the networks. Since the plotted matrices are population averages, these lines represent disconnected nodes that occur in the network of at least one subject. The percentage of subjects with disconnected nodes under each tractography and network construction setting is summarized in Table 2.

The minimum path length between two nodes is the number of edges along the shortest path. If there is no path between two nodes, the nodes are disconnected, and the path length is infinite. We calculate the minimum path length for every pair of nodes in the networks of each subject. The results are illustrated in Figure 3, where we plot the population average path length matrices at four different atlas scales with SC 10^5^ and D0. The black lines in Figure 3 represent infinite path length lines that correspond to disconnected nodes. These disconnected nodes do not form any subcomponent and have an infinite path length to every other node in the network. The appearances of the infinite path length lines demonstrate that at the particular tractography and network construction setting, there exists at least one subject among the 418 subjects with accurate coregistration of atlas regions (and a BBRegister score comparable to other successfully aligned subjects) whose network contains disconnected nodes. In this study, networks with and without disconnected nodes are called connected and disconnected networks, respectively.

Figure 3 shows that using SC 10^5^ and D0, disconnected networks occur at Scale 125 and Scale 250. The disconnected nodes only correspond to regions in the lateral orbitofrontal and medial orbitofrontal areas, which are affected by susceptibility artifact. Table 2 summarizes the percentage of subjects with disconnected networks across all tractography and network construction settings. With no grey matter dilation and a SC of 10^5^ at Scale 250, there exist 68 subjects (16.3% of the total population) with disconnected nodes. When the streamline sampling rate is increased to 10^6^, under no dilation there is only one subject whose network contains disconnected nodes at Scale 125, and 27 subjects (6.5% of the total population) have disconnected nodes at Scale 250. With grey matter dilation applied and a total SC of 10^6^, only 7 subjects remain disconnected at Scale 250, and none at other atlas scales. Since a network model with disconnected nodes is not a physically plausible model of a human brain, the result shows that for an atlas scale with as many nodes as the Scale 250 (463 nodes), a streamline sampling rate of 10^5^ is likely insufficient.

**Table 2.** The percentage of subjects whose networks contain disconnected nodes increases with brain atlas scales and decreases as the total number of streamlines or dilation increases. With SC 10^5^ and D0 at Scale 250, the percentage of networks with disconnected nodes is significantly higher than any other tractography setting. The percentage is significantly reduced by increasing streamline count to 10^6^ and applying grey matter dilation during tractography.

**Streamline Count**	**Dilation**	**Scale 33**	**Scale 60**	**Scale 125**	**Scale 250**
10^5^	0	0.00%	0.00%	0.48%	16.3%
10^5^	2	0.00%	0.00%	0.00%	3.35%
10^6^	0	0.00%	0.00%	0.24%	6.46%
10^6^	2	0.00%	0.00%	0.00%	1.67%

In the following subsections, we determine the impact of these tractography and network construction parameters on several standard graph metrics. We use assortativity, modularity, and clustering coefficients to characterize networks. Both unweighted and weighted metrics are calculated. In addition to comparing the raw values of various graph metrics, we also show that the relative values of graph metrics are dependent on tractoraphy and network construction parameters. This is accomplished by comparing the relative rank of each individual in the population distributions of graph metrics.

**Figure 4.**
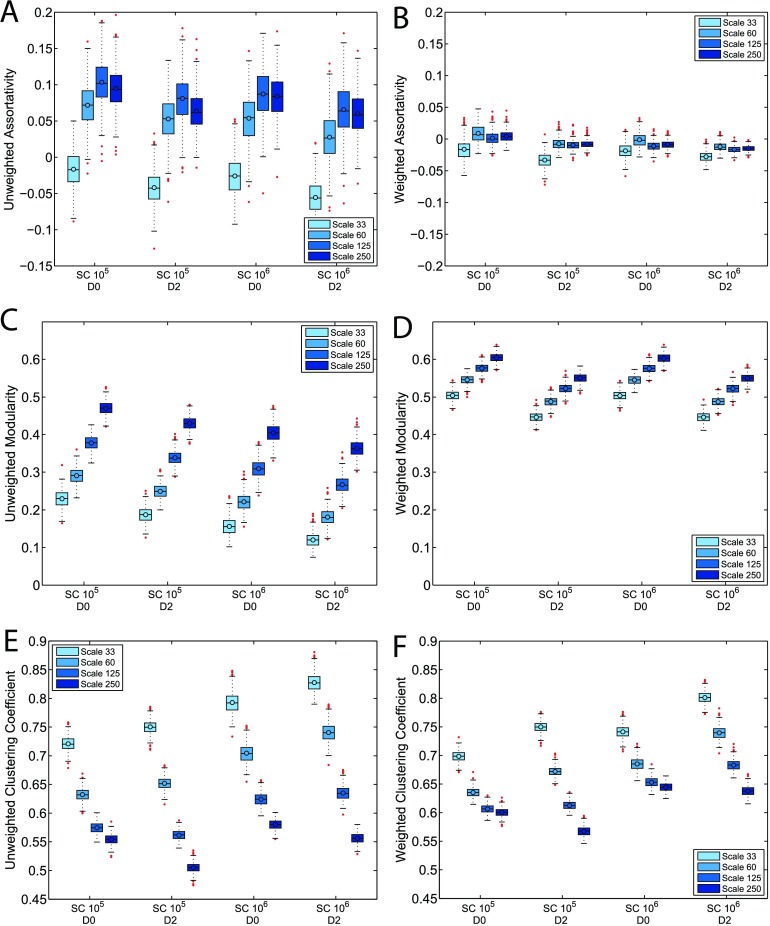
Population distributions for unweighted and weighted graph metrics with varying atlas scales, streamline count, and dilation settings. The box-and-whisker plots are defined the same way as in Figure 1. Among assortativity, modularity, and clustering coefficient, assortativity shows the least variation across atlas scales. The majority of the networks produced at Scale 33 are weakly disassortative, while most of the networks produced in other atlas scales show low but positive assortativity. Networks produced at Scale 33 also consistently have weighted clustering coefficient values that are lower than the unweighted counterparts. These results highlight the difference in network topology at Scale 33. SC and dilation settings do not affect graph metrics as significantly as atlas scale. Detailed discussions of the effects of various tractography and network construction parameters on graph metrics are included in Results and Discussion.

The graph analysis results are summarized in Figure 4, where each subfigure corresponds to one weighted or unweighted graph metric and the corresponding 16 population distributions produced under varying tractography parameters and atlas scales. The distributions are illustrated by box-and-whisker plots, following the same notation used in Figure 1.

Each subfigure of Figure 4 includes four color-coded clustered groupings, each with a set of four population distributions corresponding to increasing atlas scales from left to right. Each grouping has a corresponding streamline count and dilation setting. The first and second group have SC held constant at 10^5^; the first group corresponds to D0 and the second corresponds to D2. The third and fourth group correspond to SC fixed at 10^6^, and grey matter dilation setting D0 and D2, respectively. Comparing distributions within one clustered grouping reveals the effects of atlas scales, and the effects of streamline count and dilation can be observed by comparing distributions at the same atlas scale (indicated by color) across groupings. For example, the first and second grouping of Figure 4C compare the effects of dilation on unweighted modularity, and the first and third grouping compare the effect of streamline count.

**Figure 5.**
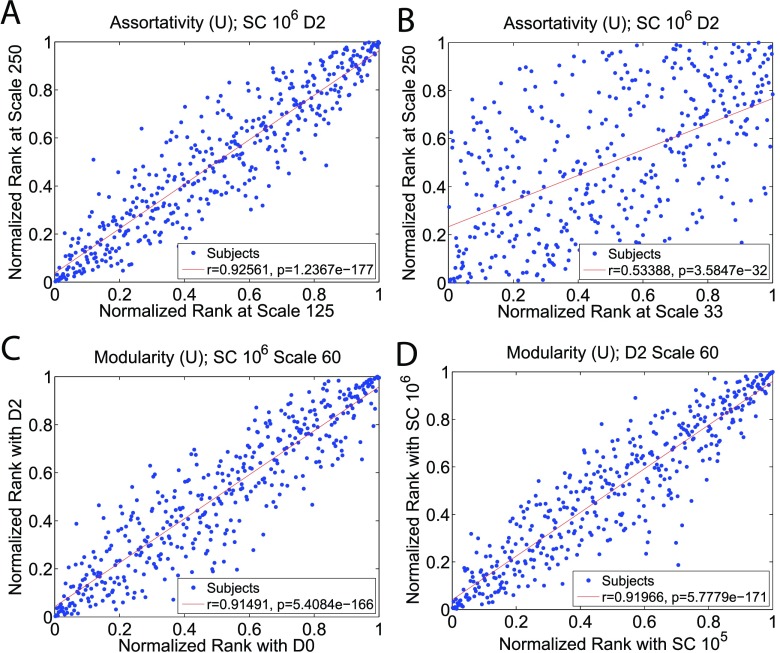
Pearson correlation tests for the normalized ranks of each individual in unweighted graph metric population distributions under varying tractography and network construction settings. In these scatter plots, each data point represents an individual’s normalized network metric ranks among the population distributions produced under two contrasting parameter values. The correlation coefficient, *r*, is also included in each subplot, with higher *r* indicating that the rank of a subject is resilient against the particular parameter value change. Figure 5A illustrates that the normalized assortativity rank of a network will remain relatively consistent when the atlas scale is changed from Scale 125 to Scale 250. However, the normalized rank can change significantly when the scale change is large. For example, Figure 5B illustrates that the assortativity rank of a subject can vary significantly at Scale 33 and Scale 250. Table 3 summarizes the rank correlations among all four atlas scales for the three graph metrics. Figure 5C and Figure 5D illustrate that grey matter dilation and streamline count have a weak effect on changing the graph metric rank of a network.

The rank comparison results are summarized in Figure 5, Table 3, and Table 4. Figure 5 illustrates four representative Pearson correlation plots in which each data point represents an individual’s normalized network metric ranks produced with two different tractography parameter values. The correlation coefficient, *r*, is also included in each subplot, with a higher *r* indicating that the rank of a subject is resilient against the particular parameter value change. Table 3 summarizes the correlation coefficients for unweighted and weighted metrics calculated with varying atlas scales, with dilation and and SC settings held constant at D2 and 10^6^, respectively. The effects of streamline count and grey matter dilation on relative ranks are presented in Table 4.

**Table 3.** Linear correlation coefficients, *r*, for unweighted and weighted graph metric rank consistency using varying atlas scales (with SC 10^6^ and D2 fixed). These correlation coefficients are derived from the Pearson correlation tests similar to Figures 5A and 5B, which measures how the rank of a subject would change in the population metric distribution when atlas scale is altered. All of the correlation coefficients reported have a corresponding *p*-value less than 0.01. In each entry, the first coefficient corresponds to the unweighted metric, and the second one corresponds to the weighted metric. With all three metrics, it is evident that the more the atlas scale is changed, the more the rank of a network changes. Different metrics are affected by scale changes differently. Clustering coefficient rank is the most unstable, while modularity rank is the most robust against atlas change. In addition, the relative ranks of weighted metrics tend to be affected more by atlas scales than unweighted metrics. Overall, these correlation coefficients illustrate that individuals are affected by atlas scale change differently.

**Assortativity**				
Scale	33	60	125	250
33	1.00			
60	0.76/0.64	1.00		
125	0.60/0.22	0.90/0.57	1.00	
250	0.53/0.13	0.80/0.46	0.93/0.86	1.00
**Modularity**				
Scale	33	60	125	250
33	1.00			
60	0.93/0.75	1.00		
125	0.86/0.68	0.93/0.76	1.00	
250	0.79/0.64	0.84/0.72	0.91/0.81	1.00
**Clustering Coefficient**				
Scale	33	60	125	250
33	1.00			
60	0.83/0.82	1.00		
125	0.54/0.51	0.83/0.80	1.00	
250	0.20/0.18	0.45/0.43	0.75/0.77	1.00

**Table 4.** Linear correlation coefficients, *r*, for unweighted and weighted graph metric rank consistency using varying dilation and streamline count settings (fixed atlas scale at Scale 60). The correlation coefficients are derived from the same procedure described in Figure 5 and Table 3. In each entry, the first coefficient corresponds to the unweighted metric, and the second one corresponds to the weighted metric. For varying dilation, the SC is fixed at 10^6^; and for varying SC, dilation is fixed at D0. Both dilation and streamline count have little effect on the unweighted modularity rank, though the weighted modularity is noticeably affected by dilation. Assortativity rank is mildly affected by dilation and streamline count. Clustering coefficient rank is robust against dilation change, and is noticeably affected by streamline count. Overall, the effect of dilation or streamline count on graph metric rank is less significant than that of atlas scale change.

	Assortativity	Modularity	Clustering Coef.
D0 vs. D2	0.83/0.81	0.91/0.66	0.90/0.86
SC 10^5^ SC 10^6^	0.84/0.77	0.92/0.87	0.74/0.77

### Assortativity

Assortativity measures the likelihood of nodes to be connected to nodes of similar degrees. Figure 4A illustrates that in unweighted networks, grey matter dilation and streamline count do not significantly affect the assortativity distributions. However, there are noticeable changes as the brain atlas scale varies. While the distributions for networks constructed at Scale 60, 125, and 250 significantly overlap, networks constructed at Scale 33 have assortativity that differs significantly from networks constructed at other atlas scales. The majority of networks constructed at atlas scales other than Scale 33 have low, yet positive, assortativity values. In contrast, the majority of networks constructed at Scale 33 are disassortative. This suggests that Scale 33 may be too coarse, producing networks with significantly different topology compared with other scales.

Figure 4B illustrates the corresponding statistics for weighted assortativity, which exhibit significantly lower variances. Scale 33 still produces networks with anomalous assortativity, though less dramatically so. Overall, weighted networks mostly have weighted assortativity values in the range between –0.05 and 0.05, which is generally interpreted as neutrally assortative (i.e., there is no bias for nodes of similar or dissimilar degree to be connected).

Atlas scale is also shown to have a significant effect on the relative ranks of individuals in the assortativity population distributions. In addition to producing significantly different raw assortativity values, networks constructed at Scale 33 tends to have noticeably different relative ranks when constructed at different atlas scales. Table 3 shows that when networks are constructed at Scale 33, their unweighted assortativity relative rank correlation coefficients are only 0.53 and 0.60 for networks constructed at Scale 250 and Scale 125, respectively. The correlations are even lower for weighted networks, with correlation coefficients of 0.22 between Scale 33 and Scale 125, and 0.13 between Scale 33 and Scale 250. The result shows that a subject can have a network that is more assortative than another subject’s network at Scale 33, yet becomes the one with the lower assortativity value when another atlas scale is used. The correlation is stronger for unweighted assortativity between Scale 60 and Scale 125 (0.90), and Scale 60 and Scale 250 (0.80). However, the correlation is still relative weak for weighted assortativity, with coefficients of 0.57 (Scale 60 vs. Scale 125) and 0.46 (Scale 60 vs. Scale 250). The correlation between Scale 125 and Scale 250 is high for both unweighted and weighted assortativity.

In comparison, streamline count and grey matter dilation do not have a strong effect on the relative ranks of assortativity values. For unweighted assortativity, streamline count and dilation result in rank correlation coefficients of 0.84 and 0.83, respectively. For weighted assortativity, the coefficients are 0.77 and 0.81, for SC and dilation, respectively.

### Modularity

Modularity quantifies the strength of division of a network into modules. Compared to assortativity, modularity shows an even more significant sensitivity to brain atlas scales, as illustrated in Figures 4C–4D. Both unweighted and weighted modularity consistently increases with atlas scales. Unweighted modularity decreases slightly with increases in streamline count and grey matter dilation. Weighted modularity is generally robust against an increase in streamline count, but decreases with grey matter dilation. Compared to unweighted modularity, weighted modularity has higher values, indicating that strongly connected nodes tend to form communities together. The weighted modularity distributions also show less variation across atlas scales, indicating that the division into communities of weighted nodes is more robust against scale changes.

Although atlas scales have a strong and consistent impact on modularity, the ranks of individuals in unweighted modularity distributions are not strongly affected by atlas scales. Table 3 shows that in the majority of the atlas scale combinations, the correlation coefficients for unweighted modularity are greater than 0.85. However, the correlation coefficients for weighted modularity are noticeably lower compared with their unweighted counterparts. Grey matter dilation and streamline count also do not alter the relative ranks of the unweighted modularity of individuals. D0 and D2 results in a correlation coefficient of 0.91, and SC 10^5^ and SC 10^6^ result in a coefficient of 0.92. The relative ranks of weighted modularity of individuals are resilient against SC changes (*r* = 0.87), but the coefficient is significantly lower for dilation changes (*r* = 0.66).

### Clustering Coefficient

Clustering coefficient measures the likelihood of the neighbors of nodes forming cliques. Figures 4E and 4F illustrate that both unweighted and weighted clustering coefficients are inversely proportional to the atlas scales. Raising streamline count increases both unweighted and weighted clustering coefficients. Dilation consistently decreases the weighted and unweighted clustering coefficients at Scale 250, while it increases them for networks constructed at Scales 33 and 60. Scale 125 does not show a general trend with dilation. Overall, the impact of dilation on network topology depends on the atlas scale.

The relationship between unweighted and weighted clustering coefficients is also scale dependent. Figures 4E and 4F illustrate that for the same set of tractography parameters and atlas scales, networks usually have higher weighted clustering coefficients than unweighted clustering coefficients, except for networks produced at Scale 33. If the weighted clustering coefficient of a network is higher than the corresponding unweighted value, triplets are more likely formed by edges with high weights. Therefore, our result implies that the low atlas scales produce networks in which triplets are formed by edges with low weights instead of high weights, again highlighting the significant effect atlas scale has on the overall network topology.

The individual ranks in clustering coefficient population distributions are heavily influenced by atlas scales. The unweighted clustering coefficient ranks of networks produced at Scale 33 have a correlation coefficient of 0.20 with those produced at Scale 250. The unweighted assortativity and modularity rank correlation coefficients for Scale 125 versus. Scale 250 are both 0.93, while for unweighted clustering coefficient it is only 0.75. The correlation coefficients for weighted clustering coefficients are very similar to unweighted clustering coefficients across all atlas scales. The low correlation coefficient shows that a comparison between the clustering coefficients of two individuals can result in drastically different conclusions depending on the atlas scale used. On the other hand, grey matter dilation and streamline count do not have strong effects on clustering coefficient ranks. D0 and D2 show correlation coefficients of 0.90 (unweighted) and 0.86 (weighted), while the coefficients between SC 10^5^ and 10^6^ are 0.74 (unweighted) and 0.77 (weighted). Clustering coefficient shows a lower correlation coefficient for varying SC compared with assortativity and modularity. However, the effect is not as drastic as atlas scales.

## DISCUSSION

A primary objective of this work is to quantify the sensitivity of inferred white matter network topology to tractography parameters and network construction methods in order to lay the foundation for constructing robust and reliable networks. The state-of-the-art imaging quality and the large sample size of the HCP provide a unique opportunity to establish a guideline for researchers to optimize the selection of tractography parameters. We also aim to serve as a reference for researchers and clinicians to better understand how their research or diagnosis conclusions may be hindered by their chosen tractography and network construction parameters.

Minimum path length reveals that, when combined with high atlas scales (highly refined regions), traditional tractography parameters and network construction settings, such as a relatively low streamline count or the absence of grey matter dilation, may lead to networks with nodes that are completely isolated. For example, Table 2 shows that if the atlas scale is changed from Scale 125 (234 nodes) to Scale 250 (463 nodes) without readjusting the streamline count (10^5^) or applying grey matter dilation, the percentage of subjects with disconnected nodes increases from 0.48% to 16.3%. The significant increase is mainly due to insufficient streamline sampling.

A network with disconnected nodes is a priori an inaccurate model; physiologically no region of the brain is isolated from other cortical regions. Although tractography-based network models can differ from the actual underlying neuronal connections, researchers and practitioners should aim to achieve the best accuracy that the DWI images and tractography algorithms allow. With a high-quality DWI dataset, the two critical factors that determine whether accurate brain network models can be constructed are the choices of tractography algorithms and tractography/network construction parameters. Current tractography methods can be largely categorized into probabilistic and deterministic algorithms. In this study we have chosen to employ a deterministic algorithm since probabilistic algorithms have been shown to produce more spurious streamlines (Maier-Hein et al., 2016). Among various deterministic algorithms, we have chosen one that is assisted by quantitative anisotropy (QA) (Yeh et al., 2013). The algorithm delivers robust performance, as proven in the ISMRM 2015 Tractometer Challenge. It is one of the best performing algorithms currently available, and it has been used in various studies (Fernández-Miranda et al., 2015; Jarbo & Verstynen, 2015; Maier-Hein et al., 2016; Meola, Comert, Yeh, Stefaneanu, & Fernandez-Miranda, 2015; Yeh et al., 2013, 2010). Despite utilizing one of the best performing algorithms and one of the highest quality DWI datasets, Table 2 illustrates that injudicious combinations of highly refined brain parcellations and low numbers of streamlines can still lead to pathologies involving isolated nodes representing grey matter regions in the constructed network model.

In many network studies, the total number of nodes is significantly higher than 463 (Irima & Van Horn, 2016; Klimm, Bassett, Carlson, & Mucha, 2014; van den Heuvel, Stam, Boersma& Pol^2008^. The streamline sampling rates are especially important for studies where high-resolution atlases are utilized. Standard tractography algorithm includes a parameter controlling the total number of streamlines (Yeh et al., 2013). For our study, we initially performed tractography with SC fixed at 10^5^. At Scale 250, there are 463 nodes. A graph with 463 nodes would require $\frac{\left(463-2)(463-1\right)}{2}+1\approx 10^{5}$ edges in order to construct a fully connected graph (Bondy & Murty, 2008). Furthermore, tractography algorithms typically generate multiple streamlines for individual node-pair connections. Therefore, while a fully connected graph with 463 nodes can have less than 10^5^ edges, at the streamline sampling rate of 10^5^, the occurrence of disconnected nodes at Scale 250 is particularly probable, and our data shown in Table 2 support this analysis. Meanwhile, with SC 10^6^, the percentage of subjects with disconnected nodes at Scale 250 decreases from 16.3% to 6.46%. A streamline count that is roughly an order of magnitude greater than the total number of node-pairs in the brain atlas is likely to capture neuronal connections more reliably than a streamline count that is less than or equal to the total number of possible connections.

In addition to the computationally demanding solution of increasing streamline count, we have also shown that grey matter dilation is an effective strategy for minimizing disconnected nodes. At Scale 250, while keeping SC at 10^5^, applying grey matter dilation reduces the percentage of subjects with disconnected nodes from 16.3% to 3.35%. When combined with an increase of SC to 10^6^, dilation further reduces the percentage of subjects with disconnected nodes to 1.67%. Although grey matter dilation is a computationally efficient solution for eliminating disconnected nodes, it has been shown that grey matter dilation in tractography can lead to spurious connections (Reveley et al., 2015). Note that the 2015 study by Reveley et al. employed grey matter seeding, while our tractography algorithm involves whole-brain seeding, with seed locations in both white and grey matter. Future studies are needed to investigate whether dilating grey matter labels would lead to spurious connections when white matter seeds are used.

Overall, Table 2 shows that a combination of increasing SC and applying grey matter dilation is very effective at reducing the likelihood of generating disconnected nodes. Our result shows the importance of selecting the proper tractography parameters in order to produce reasonable network models.

In addition to path length, we calculate weighted and unweighted assortativity, modularity, and clustering coefficients. Previous studies have used these metrics to characterize networks (Hagmann et al., 2007; Klimm et al., 2014). Although it has been shown that, for example, brain atlas scales affect graph metric values, it has not been explicitly shown that the rank orders of individuals are affected in the same manner (Zalesky et al., 2010). Therefore, in addition to studying the changes in the absolute values of graph metrics, we further differentiate our study by examining how brain atlas scales, streamline count, and grey matter dilation affect the relative values of graph metrics across individuals. The large sample size of HCP makes it feasible to infer statistically significant correlations of the ranks of the same individuals under varying tractography settings. The rank analysis illustrates the sensitivity of individual values of graph metrics relative to the population distributions. We find that the rank of each individual can be affected differently by varying tractography and network construction parameters.

Figure 4 shows that the raw values of assortativity, modularity, and clustering coefficients are heavily influenced by brain atlas parcellation scales, while grey matter dilation and streamline count have weaker, but noticeable, effects on network metrics. The strong effect of atlas scales on network metrics is reasonable, since various network metric values are heavily dependent on network sparsity (density), a fundamental property of a network. Figure 1 shows that atlas scale induces more drastic changes to network density than streamline count and grey matter dilation. However, note that network sparsity cannot be the sole contributor to changes in network metric values. For example, dilation and an increase in SC both result in networks with higher density, yet they do not always result in networks with higher clustering coefficients, as shown in Figures 4E and 4F. Yet, a decrease in atlas scale, which causes a higher network density, always results in higher clustering coefficients. In other words, changes in atlas scales, SC, and grey matter dilation may all have similar effect on network density, but they can affect network topology drastically differently.

Besides causing changes in the absolute values of various network metrics, Table 3 shows that changes in atlas scales do not simply introduce a universal factor that shifts the entire population distribution while preserving the relative rank of each individual in the distribution. It is shown that the larger the difference in atlas scales is, the more likely it is for the rank of a subject to change in the graph metric distributions. For example, the connectivity network of a subject constructed with an atlas scale might appear as highly clustered or more assortative compared with other subjects, but appear to be weakly clustered or less assortative in comparison with other subjects at higher atlas scales. Note that these effects are not introduced by resampling streamlines. These networks are constructed using the same tractograms but with different cortical labeling resolution. The inconsistency in metric rank is a direct result of atlas scale change.

Our result suggests that the effects of atlas scales on network metrics are not purely due to sparsity changes. Since sparsity and nodal degrees, two fundamental network properties, would change as the number of nodes in a network changes, it is reasonable to expect the overall distributions of graph metrics to vary with the spatial resolution of the chosen atlas scales. However, it is not trivial that each individual network would be a affected differently by atlas scales. Our findings are highly relevant to researchers and clinicians who are interested in using network diagnostics to compare the structural connectivity of the brains of experimental and control groups.

For example, assortativity reflects the resilience of a brain network to nodal disruptions that could be caused by strokes or neurodegenerative diseases that degrade certain brain regions (Rubinov & Sporns, 2010). Since assortativity measures the likelihood of nodes to be connected to nodes with similar degrees, a large and positive assortativity suggests that a network is likely to have a comparatively resilient core of mutually interconnected high-degree hubs. The rank consistency results shown in Table 3 demonstrate that a group of networks can appear to be more resilient than another group in one atlas scale but less so in a different atlas scale. Another network metric with a high level of biological relevance that can be affected by atlas scale is small-worldness. Small-worldness quantifies the balance between the functional integration and segregation of a network and is dependent on its clustering coefficient and average path length (Humphries & Gurney, 2008; Rubinov & Sporns, 2010; Watts & Strogatz, 1998). As it has been suggested that changes in small-worldness may be related to diseases such as Alzheimer’s and schizophrenia (Stam & Reijneveld, 2007), the strong dependency of the relative ranks of clustering coefficients on atlas scales may also hinder comparisons of brain networks under diseased versus normal states as characterized by small-worldness.

The rank dependence on atlas scale suggests that the relative relations between subjects may be dependent on the chosen parcellation scheme. Therefore, the diagnostic results or biological interpretation may also be dependent on the arbitrarily chosen atlas scale, which can introduce a subjective bias. Our findings show that while comparing groups of subjects in network neuroscience studies, it may be imperative to construct the networks in multiple atlas scales and observe whether the relative graph metrics are stable before concluding the characteristic differences and drawing biological interpretations.

## CONCLUSION

Tractography remains a field without a common consensus on parameter selection or a standardized pipeline. Through analysis of network metrics, we provide evidence supporting the significance of atlas scale. Both network metric values and the ranks of subjects in graph metric population distributions are sensitive to atlas scale. Through minimum path length analysis, we illustrate how imposition of high-resolution atlases mandates an increase in the total number of streamlines generated during tractography in order to produce physiologically feasible network models. Our results indicate that dilating grey matter regions during tractography and increasing the total number of streamlines are both effective in reducing the presence of disconnected nodes. Utilization of diffusion tractography to produce structural connectivity networks must consider tractography parameters in parallel with the atlas scale in order to produce network models that can accurately represent the human brain. Since the relative metric values between individuals are dependent on atlas scales, it is essential to review any biological or clinical inferences in light of the chosen atlas scale while comparing individuals.

## AUTHOR CONTRIBUTIONS

Kuang Wei: Conceptualization; Formal analysis; Investigation; Methodology; Validation; Visualization; Writing original draft; Writing review & editing. Matthew Cieslak: Conceptualization; Data curation; Methodology; Resources; Software; Writing original draft; Writing review & editing. Clint Greene: Conceptualization; Data curation; Methodology; Resources; Software; Writing original draft; Writing review & editing. Scott T. Grafton: Conceptualization; Funding acquisition; Investigation; Resources; Supervision; Writing review & editing. Jean M. Carlson: Conceptualization; Funding acquisition; Investigation; Project administration; Resources; Supervision; Writing review & editing.

## FUNDING INFORMATION

This material is supported by the David and Lucile Packard Foundation, the NFL-GE Head Health Challenge I, and the Institute for Collaborative Biotechnologies through grant W911NF-09-0001 from the U.S. Army Research Office. The content of the information does not necessarily reflect the position or the policy of the government, and no official endorsement should be inferred.

## TECHNICAL TERMS

Small-worldness: A characteristic exhibited by networks that are highly clustered yet simultaneously have small characteristic path lengths.
Rich club organization: A network organization in which well-connected nodes also connect to each other.
Network hierarchy: Networks that contain modules within modules. A module is identified as subdivisions of a network that contain densely intraconnected nodes, and each module is sparsely interconnected to other modules.
Tractography: A noninvasive and in vivo technique to reconstruct neuronal tracts in order to map the connectivity of the brain.
Diffusion-weighted imaging (DWI): A form of magnetic resonance imaging that measures the preferred orientations of the diffusion of water molecules in tissues.
Streamline: Paths reconstructed by tractography that are designed to represent the underlying neuronal connections.
Grey matter dilation: A technique in which labels of grey matter regions are artificially extended into adjacent white matter voxels. This is sometimes necessary because of the noisy signals at the grey and white matter boundaries. Dilation increases the likelihood of streamlines connecting pairs of grey matter regions when they may have terminated prematurely otherwise.
HARDI: High angular resolution diffusion-weighted imaging. An imaging technique where the number of diffusion-weighted gradient directions is significantly larger than traditional diffusion tensor imaging in order to achieve high angular resolution.
GQI: Generalized Q-sampling imaging. A model-free imaging method which samples data in the diffusion-encoding space, called q-space.
Otsu threshold: A threshold based on Otsu's method where the threshold value is determined by minimizing the sum of foreground and background spreads.
FA/GFA: Quantitative measurements of the anisotropy of water diffusion, which indicate the strength of the diffusion directionality.
ODF: Orientation distribution function, which describes the directionalities of the multimodal diffusion of water molecules in a voxel.
BBRegister: A co-registration program included with the FreeSurfer software package.
b0 DWI scan: A diffusion-weighted MR image that reflects unoriented water diffusion.
