# Supplementary material for: Sensitivity analysis of human brain structural network construction
Source: Netw Neurosci. 2017 Dec 1;1(4):446–67. doi: 10.1162/NETN_a_00025 (PMC6063716; doi:10.1162/NETN_a_00025)
Supplement: Supplementary file 1 [file netn-01-446-s001.pdf]

Wei, K., Cieslak, M., Greene, C., Grafton, S., & Carlson, J. (2017). Supporting Information for "Sensitivity analysis of human brain structural network construction." Network Neuroscience, 1(4), 446-467. https://doi.org/10.1162/netn_a_00025

Sensitivity Analysis of Human Brain Structural Network Construction

August 31, 2017

Supporting Information

SUMMARY

The Supporting Information includes supplementary figures that strengthen arguments and support discussions made in the main text.

1 List of the 49 omitted HCP subjects

In this study, we only selected subjects with a full set of 90 diffusion directions per shell. Below is the IDs of the 49 subjects who did not meet the criteria:

103515 102008 112819 113821 132118 134324 137027 137128 13793 142828 150423 150524 153833 163432 167743 171431 173132 178748 187143 192439 197550 197651 199251 208024 208428 209834 249947 329440 352738 355239 356948 382242 475855 521331 579665 585862 592455 601127 620434 644044 715041 749361 753251 792766 814649 872764 901038 951457 979984

2 Networks with SC 10^4

In addition to networks generated with SC 10^5 and 10^6, we also analyze networks that are produced with a total SC of 10^4. The analysis is not included in the main text for two reasons. First, we find that in general, the effects of SC on network metrics can be sufficiently explained by the results from networks with SC 10^5 and 10^6. Secondly, with SC 10^4, networks are significantly undersampled, resulting in a high percentage of networks being disconnected.

2.1 Disconnected Networks

Streamline Count	Dilation	Scale 33	Scale 60	Scale 125	Scale 250
10^4	0	0.24%	0.48%	8.87%	76.0%
10^4	2	0.00%	0.00%	0.72%	43.2%

Table S1: Percentage of networks with disconnected nodes at each atlas scale. At higher atlas scales, such as Scale 125 and Scale 250, significant percentages of networks generated with SC 10^4 have disconnected nodes. The high percentages indicate that a SC setting of 10^4 is too low to reconstruct enough neuronal connections at such atlas spatial resolutions.

Table S1 shows that the percentages of subjects with disconnected nodes are significantly higher when the total streamline count is only 10^4. SC 10^4 appears to be sufficient for Scale 33 and Scale 60, but insufficient at Scale 125 and Scale 250.

2.2 Network Metrics

The effect of SC on network metric distributions as shown by altering SC between 10^5 and 10^6 predicts well of how SC 10^4 would affect the metric distributions. We follow the same notation scheme for the box-and-whisker plots as shown in the main text.

Figure S1 shows that the networks produced with SC 10^4 is more sparse than networks produced with other tractography settings. Unweighted assortativity is not affected as strongly by streamline count as by dilation setting. Scale 33 still stands out in both unweighted and weighted assortativity.

The weighted assortativity with SC 10^4 and D0 shows a slightly different distribution compared to networks produced with other tractography settings. Although the majority of the networks still fall in the -0.05 to 0.05 range, thare are more outlier networks that fall outside of the range.

Unweighted modularity shows a stable trend, which tends to be influenced by network density. Weighted modularity shows the same trend revealed by networks with SC 10^5 and 10^6. Namely, SC does not have a big influence on weighted modularity. Yet, grey matter dilation tends to lower the weighted modularity.

Networks with SC 10^4 reveal a slightly different trend in both unweighted and weighted clustering coefficients. SC 10^4 is the only case where, across all scales, dilation does not increase the value of unweighted clustering coefficients. Recall that the mean network density for networks with SC 10^4 and D0 at Scale 33 is roughly 0.25. There are several tractography settings that result in a mean network density below 0.25. Namely, SC 10^4 D0 at Scale 60, SC 10^4 D0 and SC 10^5 D0 at Scale 125, and SC 10^4 D0, SC 10^5 D0, and SC 10^6 D0 at Scale 250. Figure S2e shows that for these tractography settings, applying dilation leads to decreased unweighted clustering coefficients. Given that dilation always increases network density, we deduce that when the network density is lower than ≈ 0.25, applying dilation leads to a tree like structure development in the networks, where more nodes are connected, but the neighbors of nodes are not connected.

For weighted clustering coefficient, an increase in SC always raises the coefficients. For dilation, it again appears that low density networks have a decrease in weighted clustering coefficient when dilation is applied, but there is not a clear cut off density as shown in unweighted clustering coefficient.

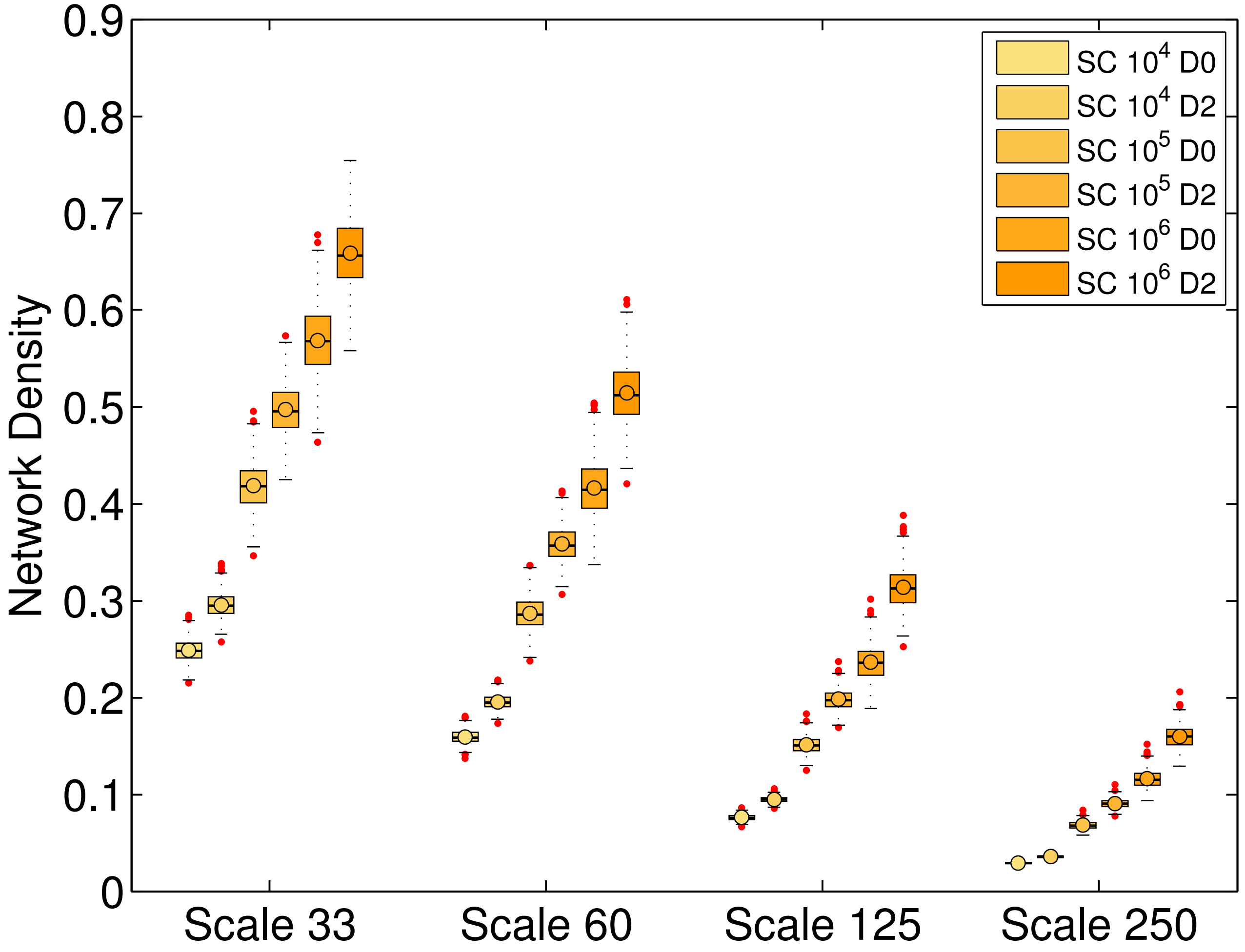

Figure S1: Network density population distributions with varying atlas resolution, dilation, and streamline count. We follow the same notation as shown in Figure 1, with the addition of population distributions produced with SC 10^4 D0 and SC 10^5 D2. As expected, networks produced with SC 10^4 show a lower network density.

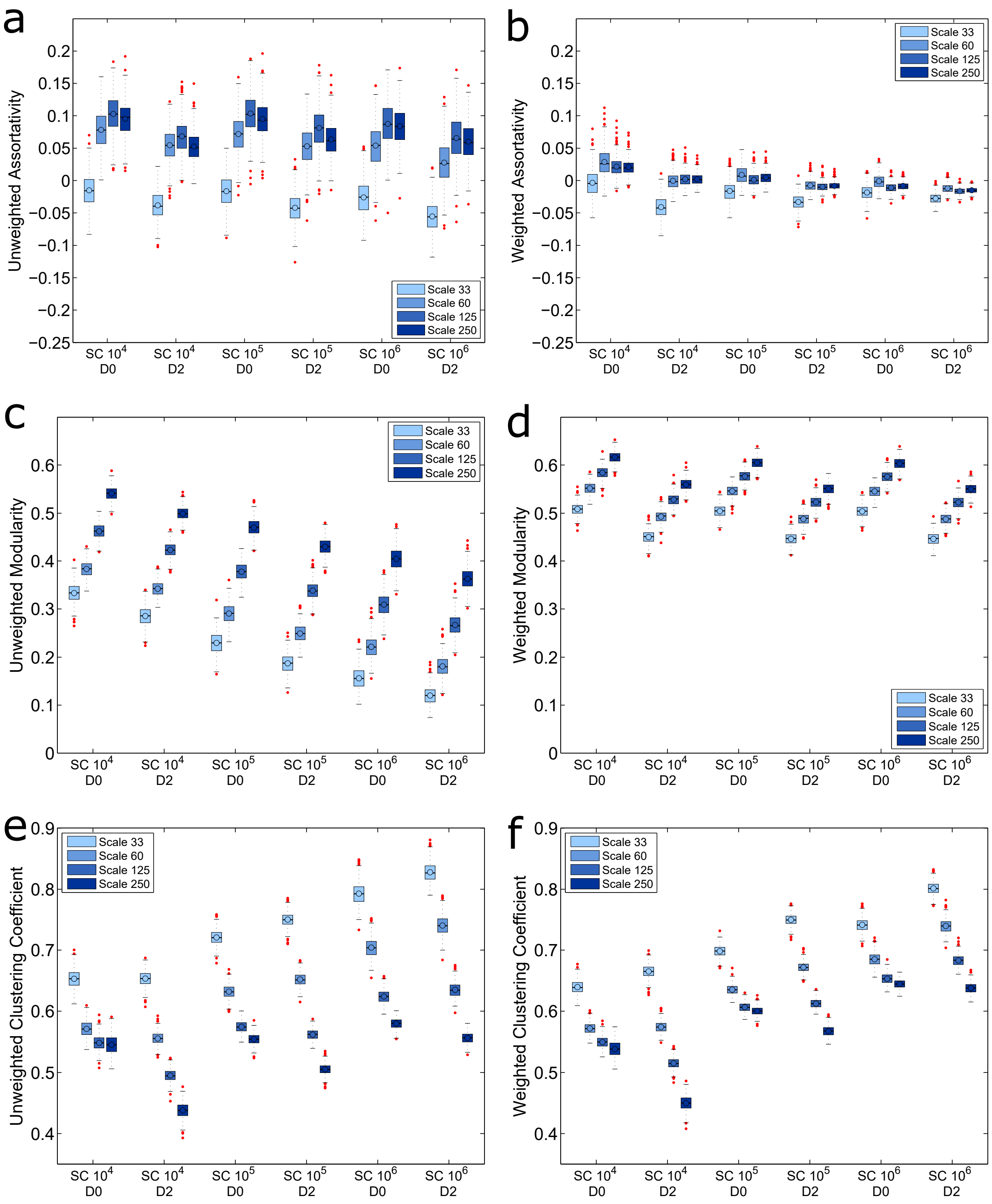

Figure S2: Population distributions for unweighted and weighted graph metrics with varying atlas resolution, streamline count, and dilation settings. The box-and-whisker plots follow the same notation as used in Figure 4, with the additional distributions correspond to SC 10^4 D0 and SC 10^4 D2. Unweighted and weighted assortativity, and weighted modularity distributions are not affected by streamline count strongly. Unweighted modularity is affected by SC which is related to density changes caused by SC changes. In Figure S2e-S2f, the additional distributions of SC 10^4 reveal an interesting correlation between network density and how dilation affects clustering coefficient. The detailed discussion is included in Section 3.2 of Supporting Information.

2.3 Individual Ranks

	Assortativity	Modularity	Clustering Coef.
SC 10^4 vs. SC 10^5	0.62	0.78	0.25
SC 10^4 vs. SC 10^6	0.42	0.70	-0.12

Table S2: Linear correlation coefficients, r, for unweighted graph metric rank consistency using varying SC settings (fixed atlas resolution at Scale 60, and fixed dilation at D2). The correlation coefficients are derived from the same procedure described in Figure 5 and Table 3. Individual ranks are affected the least in modularity population distributions. In contrast, assortativity and clustering coefficient ranks are noticeably affected by streamline count.

Table 4 shows that in the range of SC 10^5 and 10^6, SC does not affect the individual ranks in the population distributions of network metrics significantly. In contrast, Table S2 shows that the ranks of networks produced with SC 10^4 do not have high correlations with their ranks when the SC is increased to 10^5 or 10^6. For example, Figure S2a shows that SC changes do not cause changes in the overall population distributions of unweighted assortativity. Yet, Table S2 shows that the ranks of individuals in the unweighted assortativity distributions are altered when SC is increased from 10^4 to 10^5 or 10^6.

The changes in individual ranks may not be ideal since it shows that, in comparison to other subjects, an individual's structural network could be characterized drastically differently when the streamline sampling rate is altered. Recall that at Scale 125, SC 10^4 starts to show signs of severe undersampling, as indicated by the relatively high percentage of disconnected subjects in the population. Considering the issue with undersampling and changes in network metric ranks, these results indicate that even at Scale 60 (129 nodes), it may be preferable to use a SC of $\approx 10^5$, which is roughly 10 times higher than the minimum number of edges required to guarantee a fully connected network. Finding the fundamental cause of the changes in network metric ranks when SC is increased from 10^4 is beyond the scope of this study and is of interest of future research.
